# Abnormal retinal microvasculature found in active rheumatoid arthritis:a different perspective of microvascular health

**DOI:** 10.3906/sag-1806-1

**Published:** 2019-02-11

**Authors:** Hakan BABAOĞLU, Ata BAYTAROĞLU, Murat TORĞUTALP, Abdulsamet ERDEN, Sibel KADAYIFÇILAR, Umut KALYONCU

**Affiliations:** 1 Department of Internal Medicine, Faculty of Medicine, Hacettepe University, Ankara Turkey; 2 Department of Ophthalmology, Faculty of Medicine, Hacettepe University, Ankara Turkey; 3 Division of Rheumatology, Department of Internal Medicine, Faculty of Medicine, Hacettepe University, Ankara Turkey

**Keywords:** Rheumatoid arthritis, retinal vascular caliber, microvascular health, cardiovascular risk

## Abstract

**Background/aim:**

We aimed to assess the association between retinal vascular caliber (RVC) scores and disease activity in rheumatoid arthritis (RA) patients.

**Materials and methods:**

Forty-seven RA patients, 32 systemic lupus erythematosus (SLE) patients, and 45 healthy people were enrolled. RA and SLE patients were subdivided into groups according to C-reactive protein (CRP) levels. RA patients were also grouped according to Disease Activity Score-28 (DAS-28). Fundus photography was performed for all patients. RVC was summarized as the central retinal artery and vein equivalents (CRAE and CRVE).

**Results:**

Mean CRVE for RA patients was 213.3 ± 17.8 µm compared with 209.2 ± 14.1 µm for SLE and 217.5 ± 26.2 µm for the control group (P = 0.17). RVC scores did not differ between the CRP-high and CRP-low groups. As the RA disease activity increased, the widening of CRVE became more prominent and statistically significant. When the DAS-28 > 5.1 (CRVE, 220.4 (211.8–246.5) µm) group and DAS-28 ≤ 3.2 (CRVE, 214.4 (172.4–242.3) µm) group were compared, statistical significance was more pronounced (P = 0.03) than when comparing the DAS-28 > 3.2 and DAS-28 ≤ 3.2 groups (P = 0.05).

**Conclusion:**

CRVE, which reflects systemic inflammation and possibly increased cardiovascular risk, was significantly increased in active RA patients. The association between retinal venular widening and disease activity, regardless of CRP, may be a sign that RA-related inflammation may have systemic vascular effects even with normal levels of CRP.

## 1. Introduction

Cardiovascular diseases (CVDs) are the leading cause of death worldwide (1). Microvascular disease plays a role in the early development of CVDs and can be reversed. For this reason, strategies for improving microvascular health are a favorite subject for researchers (2,3). Rheumatoid arthritis (RA) is known to be associated with endothelial dysfunction and a greater risk of cardiovascular (CV) morbidity and mortality (4–6). The excess of CV morbidity and mortality in RA patients cannot be explained by traditional risk factors. There is much evidence that chronic inflammation and autoimmunity may cause this increased risk (2,4,7).

Assessment of microvascular structure is valuable in CV risk analysis in RA patients (8). Recent technological advances in high-resolution digital photography and image processing software programs have enabled the quantitative and reproducible measurement of various changes in retinal vasculature (9). The concept of retinal vascular caliber (RVC) emerged after this innovation as a noninvasive tool for assessing microcirculation. Independent of traditional risk factors, RVC widening has been found to be related to inflammation, endothelial dysfunction, and increased risk of coronary heart disease, stroke, and CV mortality (10). Epidemiological studies have shown that RVC is a cardiovascular event predictor (11–13). Demonstrating the association between RA disease activity and RVC may provide evidence of the increased CV risk in RA, which may be reversible with effective treatment (14). 

A study conducted in 2011 showed that patients diagnosed with RA according to the criteria of 1987 had wider RVC than the normal population and this widening was associated with disease activity (15). In an article published in 2016, the reduction of RVC was proved in the case of suppression of inflammation in RA patients (16).

With this background, the objective of this study was to assess RVC scores in RA patients and compare them with systemic lupus erythematosus (SLE) patients and a healthy control group. The second objective was to investigate the association and correlation between RVC scores and RA disease activity indexes at the beginning and at follow-up.

## 2. Materials and methods

### 2.1. Study population

Between July 2014 and January 2015, 49 consecutive RA patients as defined by the 2010 ACR-EULAR criteria (17) were enrolled in this prospective observational study. Thirty-two SLE patients as defined by the 1997 ACR criteria (18) and 45 healthy people who had already been referred to the department of eye diseases and had no known medical issues other than refractive error of the eyes constituted the comparison groups. Exclusion criteria were acute glaucoma, inability to provide informed consent, and ungradable retinal photographs. Two RA patients were excluded from the research because of glaucoma and ungradable retinal photographs.

### 2.2. Collecting data

Demographic data, medical history, current medications, physical measurements, and serum biochemical parameters of all participants were recorded. All traditional CVD risk factors such as diabetes mellitus, hypertension, hyperlipidemia, and smoking status were noted. Diabetes was defined as fasting serum glucose level of ≥126 mg/dL or HbA1c of ≥6.5%, or current antidiabetic drug use. Hypertension and hyperlipidemia were defined by the use of antihypertensive or antilipidemic drugs or current guideline recommendations (19,20), and patients’ statements were used to determine hyperlipidemia in the event that patients’ previous lipid levels could not be obtained. Smoking status was defined as active based on smoking within 5 years and packs per year. Body mass index (BMI) was calculated for each individual. Patients were divided into groups with BMI of 18.5–24.9 as normal, 25–29.9 as overweight, and ≥30 as obese.

### 2.3. Outcome measures

#### 2.3.1. Rheumatologic assessments

Tender Joint Count 28 (TJC), Swollen Joint Count 28 (SJC), erythrocyte sedimentation rate (ESR), C-reactive protein (CRP), and patient global assessment (visual analog scale of 0–100 mm) values were recorded at baseline. Disease Activity Score 28 (DAS-28) was calculated to assess disease activity (21,22). DAS-28 of >5.1 indicates high disease activity whereas DAS-28 of <3.2 indicates low disease activity (23). Functional status was evaluated by the Health Assessment Questionnaire-Disability Index (HAQ-DI) (24).

RA patients were invited for reevaluation within 3–6 months. Clinical and biochemical assessments and DAS-28 and HAQ-DI scores were measured and recorded again. 

RA and SLE patients with known CRP values were divided into 2 groups with a CRP cutoff value of 0.8 mg/dL for comparison (reference value).

#### 2.3.2. Retinal vascular assessments

Digital retina photographs of all individuals involved in the study were taken with a nonmydriatic 30° digital color fundus camera (Zeiss FF450 IR+) with a standardized method (9) and stored in the Zeiss Visupac System. Retinal photographs of RA patients were recorded again at the second visit.

RVC was measured by an experienced retina specialist who did not know the characteristics of the participants using the standardized computer-assisted semiautomated IVAN software (University of Wisconsin, USA). All retinal vessels within a 0.5–1 disc diameter of the optic disc margin were measured. Measurements from the six largest retinal arterioles and venules were summarized as the central retinal arteriolar equivalent (CRAE) and central retinal venular equivalent (CRVE) using the Parr-Hubbard–Knudtson formula (25) (Figure). 

The study was approved by the Hacettepe University Health Ethics Committee and was conducted in accordance with the Declaration of Helsinki. Written informed consent was obtained prior to recruitment.

**Figure 1:**
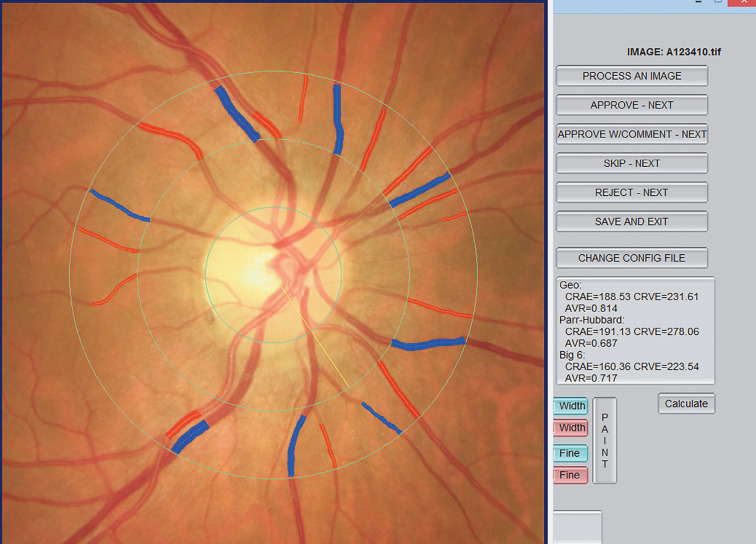
Example of calculating RVC scores with IVAN software.

### 2.4. Statistical analysis

The data were analysed with SPSS 21.0 for Windows, provided by Hacettepe University. Distribution of numerical variables was examined visually (histogram and probability graphs) and by analytical methods (Kolmogorov–Smirnov and Shapiro–Wilk tests). For univariate association, parametric continuous variables were examined with the Student t-test and nonparametric ones with the Mann–Whitney U test. ANOVA or K-independent samples tests were used according to distribution for triple group analysis. The presentation of the data was done in accordance to the distribution of variables. Normally distributed variables are presented as mean ± standard deviation while nonnormally distributed variables are presented as median (min–max). Exceptions are noted in the tables. Nominal data were compared with chi-square or Fisher tests. Correlation analyses were done with Pearson or Spearman correlation tests according to suitability. P < 0.05 was considered as a statistically significant result.

## 3. Results

### 3.1. Patients’ demographics and comparison of retinal caliber between the groups

#### 3.1.1. Study population 

We enrolled 47 RA patients comprising 39 females (83%) and 8 males. The mean age was 52.0 ± 11.9 years and the median disease duration was 8.3 years (0–30 years). Four of the RA patients (8.5%) had diabetes mellitus, 19 (40.4%) had hypertension, and 9 (19.1%) had hyperlipidemia. Nineteen (40.4%) of the RA patients had smoked previously, while 14 (29.8%) of them were actively smoking or had a history of smoking during the last 5 years. The mean BMI of the patients was 27.9 ± 5 and 29.7% of RA patients were obese. SLE patients were younger (P = 0.01). There was a statistically significant difference between the RA and SLE groups in terms of CRP (P = 0.05). Although three of the SLE patients had renal involvement in past, the glomerular filtration rate values of all patients were within the normal range at the time of the study and none of the patients had chronic renal disease (creatinine values of the control group are unknown). There was no statistical significant difference between the groups in terms of instantaneous blood pressure values. There was no difference in terms of other parameters between the groups (Table 1). 

**Table 1:** Comparison of RA, SLE, and control groups.

	RA (n = 47)	SLE (n = 32)	Control (n = 47)	P
Age, mean ± SD	52.1 ± 11.9	41.4 ± 14.5	47.2 ± 17.3	0.01
Female, n (%)	39 (83.0)	27 (84.4)	36 (80.0)	0.87
Diabetes, n (%)	4 (8.5)	1 (3.1)	5 (11.1)	0.44
Hypertension, n (%)	19 (40.4)	10 (31.3)	12 (26.7)	0.36
Dyslipidemia, n (%)	9 (19.1)	1 (3.1)	6 (13.3)	0.11
Smoking, n (%)	14 (29.8)	12 (37.5)	13 (28.9)	0.69
BMI, mean ± SD	27.9 ± 5.0a	25.8 ± 4.9b	26.2 ± 4.6	0.13
CRP, mg/dLc	0.6 (0.1–4.2)	0.4 (0.1–20.6)	n/a	0.05*

BMI: Body mass index; CRP: C-reactive protein; n/a: not available; RA: rheumatoid arthritis; SLE: systemic lupus erythematosus; ^a^: 1, ^b^: 2 missing data; ^c^: median and min–max. *: CRP value is only available for RA and SLE patients. Bold font indicates statistically significant difference.

#### 3.1.2. Rheumatologic assessments

The clinical characteristics determined at the initial evaluation of RA patients are shown in Table 2. When we classified our RA patients according to the DAS-28, 15 (32%) patients were in remission, whereas 7 (15%) had low, 15 (32%) had moderate, and 10 (21%) had high disease activity.

**Table 2:** Baseline disease activity parameters of RA patients.

Rheumatologic assessments	Results
Swollen Joint Count (28 joints), median (min–max)	0 (0–8)
Tender Joint Count (28 joints), median (min–max)	2 (0–15)
Patient Global Assessment (0–100 mm), mean ± SD	42 ± 25
DAS-28, mean ± SD	3.6 ± 1.7
HAQ-DI*, mean ± SD	0.57 ± 0.50
CRP, mg/dL, median (min–max)	0.6 (0.1–4.2)
ESR, mm/h, median (min–max)	19 (2–67)

CRP: C-reactive protein; DAS-28: Disease Activity Score-28; ESR: erythrocyte sedimentation rate; HAQ-DI: Health Assessment Questionnaire-Disability Index; *: HAQ-DI score was available for 33 patients.

#### 3.1.3. Retinal vascular assessments

Patients with RA had a mean CRAE of 147.8 ± 11.7 µm compared with 148.3 ± 12.8 µm in SLE and 147.4 ± 17.6 µm in the control group (P = 0.97). Their mean CRVE was 213.3 ± 17.8 µm compared with 209.2 ± 14.1 µm in SLE and 217.5 ± 26.2 µm in the control group (P = 0.17) (Table 3). There was no difference in retinal caliber between patients with RA and controls. Table 3 compares retinal vascular assessments of patients with RA, patients with SLE, and healthy controls. The patient population consisting of RA and SLE groups (n = 79) was dichotomized into 2 groups with a CRP cutoff value of 0.8 mg/dL. There was no difference between the CRP-high group (n = 52) and the CRP-low group (n = 27) in terms of other risk factors, SLE/RA patient populations, and CRVE and CRAE values.

**Table 3:** Retinal vascular caliber measurements of RA, SLE, and control groups participating in the study.

	RA (n = 47)	SLE (n = 32)	Control (n = 47)	P
CRAE, mean ± SD, µm	147.8 ± 11.7	148.3 ± 12.8	147.4 ± 17.6	0.97
CRVE, mean ± SD, µm	213.3 ± 17.8	209.2 ± 14.1	217.5 ± 26.2	0.17

CRAE: Central retinal arteriolar equivalent; CRVE: central retinal venular equivalent; RA: rheumatoid arthritis; SLE: systemic lupus erythematosus.

### 3.2. Association between rheumatoid arthritis disease activity and central retinal caliber 

Patients were divided into groups according to DAS-28 scores. While there was no difference in demographic or cardiovascular risk factors between the groups, it was seen that as the disease activity increased, the widening of CRVE became more prominent and statistically significant. When the DAS-28 > 5.1 (CRVE, 220.4 (211.8–246.5) µm) group and DAS-28 ≤ 3.2 (CRVE, 214.4 (172.4–242.3) µm) groups were compared, statistical significance was more pronounced (P = 0.03) than that between the DAS-28 > 3.2 (CRVE, 217.7 (160.8–247.2) µm) and DAS-28 ≤ 3.2 groups (P = 0.05). There was no difference in arteriolar caliber between patients with RA and controls in any subgroup analyses (Table 4).

**Table 4:** Retinal vascular caliber values according to various groups defined by DAS-28 score.

	DAS-28 ≤ 3.2 (n = 22)	DAS-28 > 3.2 (n = 25)	P
CRAE, µm	145.8 (120.8–169.1)	147.8 (123.3–167.6)	0.24
CRVE, µm	214.4 (172.4–242.3)	217.7(160.8–247.2)	0.05
	DAS-28 ≤ 3.2 (n = 22)	DAS-28 > 5.1 (n = 10)	P
CRAE, µm	145.8 (120.8–169.1)	151.4 (146.3–167.6)	0.12
CRVE, µm	214.4 (172.4–242.3)	220.4 (211.8–246.5)	0.03

Bold font represents statistically significant values.

### 3.3. Serial retinal vascular caliber measurements

Twenty-four (51%) of the 47 RA patients were assessed 5.2 ± 2.1 months later at a second visit. The first and second visit parameters of the patients were not statistically significantly different (Table 5). There was no significant correlation between the changes of CRAE or CRVE and DAS-28, CRP, and HAQ scores.

**Table 5:** The first and second visit parameters of RA patients.

	1st visit	2nd visit
DAS-28	3.66 (0.63–6.72)	3.22 (1.18–5.24)
CRP, mg/dL	0.54 (0.12–3.86)	0.45 (0.15–3.04)
CRVE, µm	217.9 (160.81–247.2)	215.5 (160.2–247.8)
CRAE, µm	147.1 (128.7–169.1)	145.7 (120–165.5)
HAQ-DI*	0.2 (0–1.05)	0.05 (0–0.95)

Data are presented as median (min–max). Twenty-four (51%) of the 47 RA patients attended second visits. CRP: C-reactive protein; CRAE: central retinal arteriolar equivalent; CRVE: central retinal venular equivalent; DAS-28: Disease Activity Score-28; HAQ-DI: Health Assessment Questionnaire-Disability Index. *: HAQ-DI score was available for 13 patients.

## 4. Discussion 

Few studies have been done in the field of retinal vascular health and RA. Although some researchers pay attention to this issue, information on this topic has to be increased. This study is one of the few studies examining the association of retinal vascular caliber with disease activity in patients with RA.

Our study demonstrates the significant association between retinal venular caliber and disease activity statistically. As disease activity increases, this association becomes more prominent. Our study supports previous cross-sectional studies in this respect (15,26). In addition to the current literature, our study showed that the CRVE widens as disease activity increases, independent of CRP value. Thus, even in cases of normal CRP values, RA may have systemic microvascular effects, and these effects grow while disease activity increases.

Based on our results, it is difficult to precisely say that there is a relationship between the widening of RVC and future subsequent cardiovascular events in RA patients, as the RVC change used for giving the hazard ratio in metaanalysis is large than that for our results. However, in this context, it is not appropriate to fully interpret our results according to the metaanalysis. It is known that larger venules have been associated with increased subclinical atherosclerosis, higher carotid plaque scores, and more aortic calcification and they have an independent role in predicting cardiac events in some populations, with a higher risk of stroke (12). As a result of all of this, our findings support the hypothesis that greater disease activity is associated with greater cardiovascular risk, even with normal CRP levels.

There was no difference in retinal venular caliber between the RA, SLE, and control groups. This result can be explained by the age difference between the groups, the fact that retinal venules may be wide in SLE patients (26), the lack of CRP values for the control group, and the criteria used to diagnose the RA patients. 

There are some methodological differences from other similar studies. In contrast to others, the ACR-EULAR 2010 criteria were used to diagnose RA in this study. Disease duration was similar to other studies (15,26). From this point of view, we may have a patient population with early diagnosis. Although erosions or deformities were not noted in any of these studies, there may be fewer erosions or deformities in our RA patients. Clinical disease scoring methods are also different. In one study (16), DAS-28-CRP was used, whereas in another study only CRP was used (26). These factors may be the cause of minor differences between the results of studies.

In one of the previous studies (26), a CRP–CRVE association was shown. In that study, patients were dichotomized into groups by a CRP cutoff value of 15 mg/L. When we used this level for our patient population, only 12 patients had a CRP value greater than 15 mg/L. We may not have found a CRP–CRVE association because of the distributional variability of CRP values between these studies. 

One of the few studies (16) on this subject showed that there was no difference in RVC during follow-up in stable-low inflammation RA patients, similar to our results.

The strength of our study includes the fact that it is the first study comparing RA to another rheumatologic disease and employing the most recent diagnostic criteria. A follow-up study used a highly reproducible method to assess retinal vessel size and demonstrated that RVC did not change in stable patients. This result also indicates that the measurement-bound bias is minimized. Most importantly, this is the first study to demonstrate that CRVE is related to disease activity regardless of CRP level.

The limitations of the study were being cross-sectional and observational; a small sample size for subgroup analysis; a lower number of RA patients attending the second visits; not knowing the CRP, creatinine value, and lipid profile of the control group; not knowing the lipid profiles at the time of retinal examination; and not knowing how many patients were under effective treatment.

Although retinal vascular caliber measurement is a newly defined method, the number of studies on this topic is constantly increasing. In these studies, RVC widening has been shown to be an important clue in determining cardiovascular risk and predicting CVS events (11–13). Individuals with larger RVC were found to have a higher risk of stroke, coronary heart disease, and mortality (10). It is known that CV mortality is increased in RA patients, and epidemiological studies show that this mortality increase is not fully explained by classical risk factors (4). Briefly, CRVE, which reflects systemic inflammation and possibly increased CV risk, was significantly increased in active RA patients. The association between retinal venular widening and disease activity regardless of CRP may be a sign that RA-related inflammation may have systemic vascular effects even with normal levels of CRP.

## Acknowledgments

We thank Dr Nicola Ferrier of the University of Wisconsin for permission to use the IVAN software.
